# Blood routine risk factors for coronary artery aneurysm in infants younger than 8 months with Kawasaki disease

**DOI:** 10.1186/s12887-021-03083-3

**Published:** 2022-01-07

**Authors:** Ge Haiyan, Lai Jianming, Tong Suqian, Qu Dong, Liu Shuang, Zhang Jin

**Affiliations:** 1 Department of Critical Care Medicine, Children’s Hospital, Capital Institute of Pediatrics, Beijing, 100020 China; 2 Department of Rheumatology, Children’s Hospital, Capital Institute of Pediatrics, Beijing, 100020 China; 3 Department of Cardiology, Guiyang Children’s Hospital, Guiyang Maternal and Child Health Hospital, Guiyang, 550003 Guizhou Province China

**Keywords:** Kawasaki disease, Infants, Coronary artery aneurysm, Routine blood tests

## Abstract

**Objective:**

The aims of this study were to characterize the evolution of routine blood values within the first 10 days of illness and coronary artery outcome in infants < 8 months with Kawasaki disease (KD) and to identify risk factors for coronary artery aneurysm (CAA).

**Methods:**

Laboratory data, clinical features and coronary artery outcomes from 78 infants < 8 months old and 86 patients between 8 months and 7 years old were retrospectively analyzed. Logistic regression analysis was conducted to evaluate the potential risk factors for CAA.

**Results:**

Infants < 8 months old were more likely to present with incomplete KD (37.2% vs 4.7%, *P* < 0.001), erythema and induration at the BCG inoculation site (24.4% vs 3.5%, *P* < 0.001) and CAA (47.4% vs 15.1%, *P* < 0.001) even with timely diagnosis and treatment with intravenous immunoglobulin (IVIG) compared with patients ≥8 months old. Clinical feature related to diagnostic criteria for KD including bilateral conjunctival injection, oral changes, unilateral cervical lymphadenopathy and extremity changes were less common in the younger group. During the acute phase, the percentage neutrophils and neutrophil to lymphocyte ratio [NLR] peaked on median illness day 3, followed by white blood cell (WBC) and CRP on median illness day 4, hemoglobin on median illness day 7 and platelet count on median illness day 9. CAA occurred on median illness day 6 and regressed on median illness day 28. Multivariate logistic regression analysis revealed that the peak percentage neutrophils (odds ratio [OR] per 0.1: 1.597, 95% confidence interval [CI]: 1.041–2.452, *P* = 0.032) and the peak platelet count (OR per 10 × 10^9^/L: 1.029, 95% CI: 1.004–1.055, *P* = 0.024) were independent risk factors for CAA. Hemoglobin on the 5th day was associated with persistent CAA at 1 year after KD onset.

**Conclusion:**

Factors associated with CAA include a high peak percentage neutrophils, increased peak platelet count, and reduced hemoglobin within 4–6 days during the acute phase of KD. Therefore, this population should receive primary therapy with IVIG and adjunctive anti-inflammatory medications.

## Introduction

Kawasaki disease (KD) is an acute systemic vasculitis of unknown cause that preferentially affects the coronary artery [1]. The most frequent age at onset of KD ranges from 9 to 11 months, and an onset age younger than 6 months is relatively infrequent [2]. Infants, especially those younger than 6 months, are also reported to have a higher prevalence of incomplete KD, coronary artery aneurysms (CAAs) and adverse cardiac events [3–6]. Acute KD patients at high risk for the development of CAA may benefit from primary therapy with IVIG and adjunctive anti-inflammatory medications, as outlined by the 2017 AHA guidelines [1]. Therefore, early identification of infants with KD at high risk of CAA and the provision of timely primary treatment with adjunctive therapies are very important to improve the prognosis of infants with KD. It has been reported that peripheral blood cell parameters are not only helpful to diagnose incomplete KD but also indicators of the risk of CAA among KD patients [1, 5]. However, the dynamic change of these laboratory values in infants with KD during the acute phase has rarely been reported. The aims of this study were to characterize the dynamic change of laboratory values within the first 10 days of illness and the coronary artery outcome in infants younger than 8 months with KD, and to describe the use of laboratory data to identify risk factors for coronary aneurysms in this population.

## Materials and methods

We retrospectively enrolled 78 consecutive infants younger than 8 months and 86 patients between 8 months and 7 years who were treated for KD and received intravenous immunoglobulin (IVIG) (2 g/kg × 1 day or 1 g/kg × 2 days) therapy within the first 10 days of illness at Children’s Hospital, Capital Institute of Pediatrics. Seventy patients were treated at the Department of Critical Care Medicine between October 1, 2015 and December 31, 2020, while the remaining patients were treated at the Department of Rheumatology between March 1, 2019 and December 31, 2020. Patients with congenital heart disease and patients with incomplete data were excluded from the study. We collected clinical data, including age, illness day at diagnosis (illness day 1 = first day of fever), response to IVIG therapy, complete blood count (white blood cell [WBC] count, percentage neutrophils, percentage lymphocytes, neutrophil to lymphocyte ratio [NLR], hemoglobin, platelet count), plasma concentrations of C-reactive protein (CRP) and coronary artery status. Complete blood count and CRP were collected and analyzed at 2 time points: 1st day (illness days 1–2) and 5th day (illness days 4–6). For patients with multiple laboratory values within a time interval, only the laboratory values on the 1st and 5th days of illness were analyzed. The maximal laboratory value, defined as the highest complete blood count and CRP values at any time point during the first 10 days after fever onset, was also analyzed.

Complete KD was defined as fever ≥5 days and ≥ 4 of the following clinical signs: Bilateral conjunctival injection, polymorphous skin rash, changes in the lips or oral mucosa, changes (edema/redness/peeling) of the extremities, and unilateral cervical lymphadenopathy. Patients with fever ≥5 days plus < 4 criteria with or without echocardiogram abnormalities were classified as having incomplete KD. IVIG resistance was defined as recrudescent or persistent fever at least 36 h after completion of the first IVIG infusion [1]. IVIG-resistant patients were given additional IVIG doses and steroids.

Echocardiography was performed at the time of initial diagnosis and at 1–2 weeks, 3–4 weeks and 6–8 weeks after KD onset. In addition, patients with coronary artery damage underwent echocardiograms every one to 3 months depending on the severity of CAA. Patients were classified as having normal (< 2.5 standard deviations [Z-score] from the mean, normalized for body surface area) or aneurysmal (Z ≥ 2.5) coronary arteries on the basis of the maximal internal diameters of the following segments measured by echocardiography during the first 6 weeks after fever onset: Left main coronary artery, proximal and distal left anterior descending artery (LAD), left circumflex, and right coronary artery (RCA) (proximal, middle, and distal). The Z-max was defined as the largest Z-score for any coronary artery segment on each echocardiogram at any time point during the first 6 weeks after fever onset. Persistent CAA was defined as a Z-score of ≥2.5 for the maximal internal diameters of the proximal LAD and proximal RCA on echocardiograms at 12 months after KD onset. CAA regression was defined as a Z-score < 2.5 on echocardiograms during the follow-up period. Z-scores were calculated using the Dallaire equations (https://www.pedz.de/en/welcome.html) [7]. The study was approved by the regional ethics committee.

### Statistical analyses

All continuous variables are expressed as the mean ± standard deviation or the median with interquartile range (IQR). The primary comparisons were made between infants < 8 months old and patients ≥8 months old, and the comparisons of the clinical and laboratory data between infants < 8 months old with and without CAA were also made. Comparisons between groups were performed with an unpaired Student’s t test or Mann-Whitney U test. Categorical variables are expressed as numbers and percentages and were compared using the chi-square test. After baseline comparisons were performed, the variables that were found to differ between groups were then included in a binary logistic regression analysis to control for their effects. Receiver operating characteristic (ROC) curves and the areas under the curves (AUCs) were constructed to compare the predictive value of laboratory indicators for persistent CAA. Statistical analysis was performed using SPSS software (Version 20.0. IBM Corp, Armonk, NY). Statistical significance was defined as *P* < 0.05 (two-sided).

## Results

### Patient characteristics

The median age of these patients at the time of diagnosis of acute KD was 0.85 years (IQR: 0.22–2.33 years), and 96 (58.5%) were boys. The median duration of fever before the first IVIG treatment was 5 days (IQR: 5–7 days). Of the patients in this study, 150 (91.5%) received a single course of IVIG treatment, 14 (8.5%) were IVIG resistant and received IVIG retreatment, and 9 (5.5%) patients received steroids. Even with timely diagnosis and treatment with IVIG, the incidences of CAA and persistent CAA at 1 year after KD onset were 30.5% (50/164) and 5.5% (9/164), respectively.

Infants < 8 months old were more likely to have incomplete KD (37.2% vs. 4.7%, *P* < 0.001), erythema and induration at the BCG inoculation site (24.4% vs. 3.5%, P < 0.001) and CAA (47.4% vs. 15.1%, *P* < 0.001) compared with patients ≥8 months old. Clinical feature related to diagnostic criteria for KD including bilateral conjunctival injection, oral changes, unilateral cervical lymphadenopathy and extremity changes were less commonly noted in the younger group. No significant difference was found regarding the gender, skin rash, IVIG resistance and persistent CAA at 1 year after KD onset between the two age groups (Table 1).

**Table 1:** Clinical characteristics of the study population

Characteristic	< 8 months old (*N* = 78)	≥ 8 months old CA (*N* = 86)	*P*-value
Age (months)	2.5 (1.8; 4.6)	27.5 (17.0; 36.0)	< 0.001
Male sex	46 (59.0%)	50 (60.5%)	0.914
Incomplete KD	29 (37.2%)	4 (4.7%)	< 0.001
Erythema and induration at the BCG inoculation site	19 (24.4%)	3 (3.5%)	< 0.001
Bilateral conjunctival injection,	57 (73.1%)	82 (95.3%)	< 0.001
Oral changes	60 (76.9%)	81 (94.2%)	0.001
Unilateral cervical lymphadenopathy	52 (66.7%)	81 (94.2%)	< 0.001
Extremity changes	46 (59.0%)	69 (80.2%)	0.030
Skin rash	59 (37.2%)	66 (4.7%)	0.868
CAA	37 (47.4%)	13 (15.1%)	< 0.001
Persistent CAA	7 (9.0%)	2 (2.3%)	0.128
Days of fever before IVIG infusion	5 (4;6)	6 (5; 7)	< 0.001
IVIG resistance	9 (11.5%)	5 (5.9%)	0.191
Peak WBC count (10^9^/L)	18.16 (14.45; 23.88)	16.71 (14.13; 20.0)	0.156
Peak percentage neutrophils	0.65 (0.58; 0.74)	0.77 (0.70; 0.82)	< 0.001
Peak NLR	2.74 (1.85; 3.81)	4.61 (3.16; 6.77)	< 0.001
NLR after IVIG	0.59 (0.32; 1.30)	0.70 (0.42; 1.53)	0.316
Peak hemoglobin (g/L)	85.95 ± 10.11	103.44 ± 10.89	< 0.001
Peak platelet count (10^9^/L)	693.0 (572.0; 832.5)	443.5 (367.3; 538.8)	< 0.001
Peak CRP (mg/L)	84.0 (50.5; 113.2)	70.6 (35.9; 118.4)	0.140
Laboratory data on the 1st day			
WBC count (10^9^/L)	13.16 (9.21;17.05)	14.80 (12.44; 18.35)	0.028
Percentage neutrophils	0.62 (0.49; 0.66)	0.72 (0.65; 0.80)	< 0.001
NLR	2.11 (1.37: 2.86)	3.53 (2.66; 5.95)	< 0.001
Hemoglobin (g/L)	113.00 (105.00; 126.00)	116.67 ± 14.62	0.012
Platelet count (10^9^/L)	336.5 (255.5; 414.0)	257.0 (222.0; 315.0)	< 0.001
CRP (mg/L)	39.5 (15.5; 66.6)	36.0 (18.0; 56.5)	0.739
Laboratory data on the 5th day			
WBC count (10^9/^L)	14.08 (9.46; 18.33)	13.64 (10.95; 16.39)	0.952
Percentage neutrophils	0.50 (0.34; 0.67)	0.70 (0.61; 0.77)	< 0.001
NLR	1.38 (0.67; 2.88)	3.38 (2.01; 5.30)	< 0.001
Hemoglobin (g/L)	100.00 (88.00; 108.00)	113.00 (104.50; 119.00)	< 0.001
Platelet count (10^9^/L)	404.0 (293.0; 566.0)	303.0 (257.8; 370.0)	< 0.001
CRP (mg/L)	50.0 (34.7; 96.7)	58.2 (28.8; 109.0)	0.662

### Laboratory data

Evaluation of the laboratory values by day of illness for the patients with KD showed that the WBC, percentage neutrophils, NLR, hemoglobin, platelet and CRP continued to rise or decline, peaking on different days during the acute phase. Peak values were 17.83 (IQR: 14.30–20.63) × 10^9^/L for WBC on median illness day 4 (IQR: 2–6), 0.73 (IQR: 0.64–0.79) for percentage neutrophils on median illness day 3 (IQR: 2–5), 3.65 (IQR: 2.44–5.60) for NLR on median illness day 3 (IQR: 2–5), (95.2 ± 13.7) g/L for hemoglobin on median illness day 7 (IQR: 5–9), 553.0 (IQR: 424.0–715.0) × 10^9^/L for platelet count on median illness day 9 (IQR: 8–10), and 73.4 (IQR: 44.0–114.0) mg/L for CRP on median illness day 4 (IQR: 3–5). In addition, the NLR after initial IVIG administration (median, 2 days after IVIG) was 0.65 (IQR: 0.35–1.31). Infants < 8 months old had a lower WBC count on the 1st day, percentage neutrophils, NLR, hemoglobin, and a higher platelet count at any time point, compared with patients ≥8 months old. There was no difference in the peak days of any laboratory parameter, WBC count on the 5th day, peak WBC count, and CRP at any time point by age cohort (Table 1).

### Echocardiography measurements

During the acute phase of the illness, all of the enrolled patients’ electrocardiograms showed normal left ventricular ejection fraction before IVIG. Compared with patients in the older group, a larger proportion of infants < 8 months old had CAA (33.3% Z-score ≥ 2.5 to < 5, 11.5% Z-score ≥ 5 to < 10, 2.6% Z-score ≥ 10 vs. 12.8% Z-score ≥ 2.5 to < 5, 2.3% Z-score ≥ 5 to < 10, 0.0% Z-score ≥ 10, *P* < 0.001). CAA occurred on median illness day 6 (IQR: 5–8, range 1–33). Twenty-five patients (15.2%) developed CAA in the bilateral coronary arteries, 16 (9.8%) developed CAA in the left coronary artery, and 9 (5.5%) developed CAA in the RCA. The locations of CAA included the LAD in 28 (17.1%), the left circumflex in 10 (6.1%), the proximal RCA in 34 (20.7%), the middle RCA in 14 (8.5%), and the distal RCA in 8 (4.9%). The median follow-up time for the entire cohort was 510 days (IQR 391–764 days). CAA regression occurred at a median time of 28 days (IQR: 15–115 days) in 41 patients (25.0%). However, nine patients (5.5%) including 7 patients < 8 months old and 2 patients ≥8 months old had persistent CAA at 1 year after KD onset. No significant difference was found regarding the locations of CAA and the incidence of persistent CAA between the two age groups.

### Risk factors associated with coronary aneurysms

Patients with CAA were younger (0.26 [IQR: 0.16–0.81] years vs. 1.5 [IQR: 0.38–2.92] years, *P* < 0.001) and had a higher platelet count on the 1st day (345.0 [IQR: 245.0–414.0] × 10^9^/L vs. 269.0 [IQR: 230.0–333.0] × 10^9^/L, *P* = 0.009), platelet count on the 5th day (404 [IQR: 257.5.0–575.0] × 10^9^/L vs. 321.5 [IQR: 268.5–413.5] × 10^9^/L, *P* = 0.033), peak platelet count (674.0 [IQR: 554.5–877.5] × 10^9^/L vs. 501.5 [IQR: 400.0–644.0] × 10^9^/L, *P* < 0.001), peak WBC count (19.57 [IQR: 15.50–24.61] × 10^9^/L vs. 17.06 [IQR: 13.92–19.82] × 10^9^/L, *P* = 0.01), a lower hemoglobin on the 5th day (101.06 ± 17.11 g/L vs. 108.30 ± 14.51 g/L, *P* = 0.007) and peak hemoglobin (88.45 ± 13.08 g/L vs. 98.07 ± 12.93 g/L, *P* < 0.001) as compared to patients without CAA. There was no difference in the WBC counts on the 1st and 5th day, percentage neutrophils, NLR and CRP at any time point between patient with and without CAA. Multivariate logistic regression analysis showed that the independent risk factors for CAA were age (odds ratio [OR] per 1 year: 0.630, 95% confidence interval (CI): 0.418–0.950, *P* = 0.027) and peak platelet count (OR per 10 × 10^9^/L: 1.033, 95% CI: 1.012–1.054, *P* = 0.002).

Table 2 describes the clinical and laboratory data of 37 and 41 infants < 8 months with CA Z-scores of ≥2.5 and < 2.5, respectively, during the acute KD phase. Univariate analysis revealed four potential risk factors associated with CAA, including peak percentage neutrophils, peak NLR, peak platelet count, and WBC count on the 1st day. Multivariate logistic regression analysis was conducted to evaluate the independent effects of the above associated factors on the development of CAA in the younger age group. Peak percentage neutrophils (OR per 0.1: 1.597, 95% CI: 1.041–2.452, *P* = 0.032) and peak platelet count (OR per 10 × 10^9^/L: 1.029, 95% CI: 1.004–1.055, *P* = 0.024) were independent risk factors for CAA.

**Table 2:** Characteristics of infants < 8 months with and without CAA during the acute phase of KD

Characteristic	CAA (*N* = 37)	Normal CA (*N* = 41)	*P*-value
Age (months)	2.4 (1.8;3.7)	3.0 (2.0; 4.9)	0.156
Male sex	19 (51.4%)	27 (65.9%)	0.194
Incomplete KD	15 (40.5%)	14 (34.1%)	0.560
Days of fever before IVIG infusion	5 (4; 6)	5 (4; 6)	0.885
IVIG resistance	7 (18.9%)	2 (4.9%)	0.113
Peak WBC (10^9^/L)	20.64 (15.09; 27.52)	17.42 (13.40; 19.33)	0.052
Peak percentage neutrophils	0.69 ± 0.12	0.61 ± 0.13	0.007
Peak NLR	3.08 (2.37; 5.15)	2.46 (1.46; 3.57)	0.013
NLR after IVIG	0.58 (0.32; 1.41)	0.67 (0.30; 1.24)	0.673
Peak hemoglobin (g/L)	83.94 ± 10.26	87.71 ± 9.77	0.104
Peak platelet count (10^9^/L)	793.0 (576.5; 962.5)	659.0 (562.0;758.5)	0.015
Peak CRP (mg/L)	75.5 (50.0; 135.3)	84.0 (59.5; 104.7)	0.702
Laboratory data on the 1st day			
WBC count (10^9^/L)	15.48 (10.78;18.66)	12.50 (9.00; 14.88)	0.045
Percentage neutrophils	0.61 ± 0.13	0.55 ± 0.17	0.113
NLR	2.11 (1.47;3.05)	2.10 (1.18; 2.75)	0.243
Hemoglobin (g/L)	116.60 ± 15.54	115.53 ± 14.21	0.757
Platelet count (10^9^/L)	366.2 ± 146.5	320.2 ± 97.0	0.106
CRP (mg/L)	43.3 (15.3; 68.0)	29.0 (15.1; 61.5)	0.381
Laboratory data on the 5th day			
WBC count (10^9/^L)	14.44 (9.76; 20.33)	12.69 (8.73; 17.25)	0.070
Percentage neutrophils	0.55 ± 0.20	0.47 ± 0.19	0.095
NLR	2.03 (0.62; 3.33)	1.26 (0.66; 2.00)	0.073
Hemoglobin (g/L)	97.78 ± 17.30	101.78 ± 16.55	0.303
Platelet count (10^9^/L)	452.7 ± 200.6	398.3 ± 143.8	0.172
CRP (mg/L)	45.85 (33. 5; 96.5)	59.0 (36.8; 98.0)	0.614

Table 3 shows the clinical and laboratory data of 7 patients with persistent CAA and 71 patients with Z-scores < 2.5 at 1 year after KD onset in the younger group. Univariate analysis revealed two potential risk factors associated with persistent CAA: Hemoglobin on the 5th day and peak hemoglobin. The ROC curve for the prediction of a Z-score < 2.5 at 1 year after KD onset based on hemoglobin on the 5th day was conducted. The AUC was 0.829 (95% CI 0.711–0.946, *P* = 0.008). As with the optimal cutoff value of 89.5 g/L, hemoglobin on the 5th day had 77.5% sensitivity (83.3% specificity) for a Z-score < 2.5 at 1 year after KD onset.

**Table 3:** Characteristics of infants < 8 months with and without persistent CAA at 1 year after KD onset

Characteristic	Persistent CAA (*N* = 7)	Normal CA (*N* = 71)	*P*-value
Age (months)	2.5 (1.8; 3.6)	2.5 (1.8; 4.7)	0.820
Male sex	3 (42.9%)	43 (60.5%)	0.613
Incomplete KD	3 (42.9%)	26 (36.6%)	1.000
Days of fever before IVIG infusion	5 (4;10)	5 (4; 6)	0.323
IVIG resistance	2 (28.6%)	7 (9.9%)	0.391
Peak WBC count (10^9^/L)	19.79 (15.26; 39.29)	18.16 (14.44; 23.73)	0.392
Peak percentage neutrophils	0.73 ± 0.12	0.64 ± 0.13	0.138
Peak NLR	3.41 (2.53; 9.11)	2.68 (1.71; 3.80)	0.174
NLR after IVIG	0.60 (0.30; 2.57)	0.59 (0.31; 1.28)	0.893
Peak hemoglobin (g/L)	77.83 ± 9.06	86.63 ± 9.95	0.040
Peak platelet count (10^9^/L)	785.7 ± 112.4	706.7 ± 221.5	0.392
Peak CRP (mg/L)	125.5 (51.8; 162.5)	82.0 (50.0; 110.0)	0.100
Laboratory data on the 1st day			
WBC count (10^9^/L)	16.58 (11.81;19.36)	13.13 (9.05; 16.83)	0.184
Percentage neutrophils	0.63 ± 0.17	0.57 ± 0.15	0.456
NLR	2.76 (1.45: 4.43)	2.04 (1.35; 2.86)	0.350
Hemoglobin (g/L)	108.67 ± 15.67	116.67 ± 14.62	0.205
Platelet count (10^9^/L)	364.7 ± 115.5	339.4 ± 124.8	0.634
CRP (mg/L)	46.00 (14.0; 86.2)	37.0 (15.7; 64.8)	0.700
Laboratory data on the 5th day			
WBC count (10^9/^L)	14.52 (8.84; 17.42)	14.08 (9.46; 18.50)	0.992
Percentage neutrophils	0.52 ± 0.24	0.50 ± 0.20	0.837
NLR	2.18 (0.50; 4.63)	1.37 (0.66; 2.77)	0.610
Hemoglobin (g/L)	83.50 ± 9.61	101.230 ± 16.70	0.012
Platelet count (10^9^/L)	424.0 ± 284.7	423.7 ± 164.2	0.997
CRP (mg/L)	82.3 (46.3; 125.8)	47.1 (34.4; 95.5)	0.215

Multivariate logistic regression analysis was also conducted to evaluate the independent effects of peak percentage neutrophils, peak NLR, WBC count on the 1st day and variables that differed between groups with and without CAA on the development of CAA in the older age group. Peak platelet count (OR per 10 × 109/L: 1.046, 95% CI: 1.002–1.092, *P* = 0.042) was an independent risk factors for CAA.

## Discussion

The data of this study showed that 37.2% of infants younger than 8 months had incomplete KD, 47.4% of these infants had CAA, and 9.0% had persistent CAA at 1 year after KD onset. Consistent with the results described in this study, Salgado et al. [4] found that infants < 6 months old with acute KD are more likely to present with incomplete KD and develop CAA despite timely treatment with IVIG within the first 10 days. As KD is a form of systemic vasculitis, it may lead to dynamic changes in peripheral blood cells, characterized by an elevated WBC count, percentage neutrophils, platelet count, and anemia over time [8]. As CAA most often occurs within 8 days after the onset of fever [9], we focused on the dynamic nature of peripheral blood cells over 10 days and found that WBC, percentage neutrophils, platelet count and hemoglobin peaked on different days after KD onset, suggesting that peripheral blood cell parameters played significantly different roles in the dynamic process of KD inflammation. Further, we also found that peak percentage neutrophils was significantly associated with the risk of CAA in infants younger than 8 months, which was not suitable for patients ≥8 months old. Last, another finding was that hemoglobin on the 5th day was closely related to persistent CAA.

These data showed that the median peaks of WBC and percentage neutrophils were at 3–5 days of illness onset. Furthermore, the peak percentage neutrophils was an independent predictor of CAA. Previous studies showed that the number of circulating functionally activated neutrophils increased and produced excessive reactive oxygen species and elastase, which may contribute to the formation of coronary artery lesions in patients with KD during the acute phase [10, 11]. Takahashi et al. [12] also reported that numerous neutrophils were identified in the coronary artery lesions of patients who died 10 days after KD onset, and these high numbers of neutrophils were involved in the initial damage to the coronary artery during the acute phase of KD. Ha et al. [13] reported that KD patients with CAA had a higher acute NLR than patients without CAA, and we also found a significant difference in the peak NLR between infants younger than 8 months with and without CAA in this study. Therefore, the domination of leukocytes by neutrophils plays an important role during the early stage of KD and is significantly associated with the risk of CAA.

In this study, the platelet count peaked within 8–10 days of illness and had independent predictive value for CAA. This was consistent with the findings of Ueno et al. [14], who reported that platelet-neutrophil aggregates were significantly associated with pathological developments of CAA in KD. During the acute phase of KD, coronary artery injury leads to increased platelet activation and an increase in platelet-neutrophil aggregates, resulting in amplified vascular inflammation and tissue injury. Prednisolone could inhibit platelet-neutrophil aggregate-induced amplified vascular inflammatory activation [14, 15]. Therefore, platelets had an important effect on coronary vascular inflammation after initial neutrophil infiltration, especially on illness days 8–10, and were predictive of CAA development.

Another significant finding in our study was that the median illness day of the lowest hemoglobin level was 7 and that hemoglobin on the 5th day was closely related to persistent CAA at 1 year after KD onset. Kosaka et al. [16] also found that lower acute age-adjusted hemoglobin levels were correlated with the risk of coronary dilation in patients with incomplete KD. Tremoulet et al. [8] revealed that anemia was normocytically normochromic and resolved spontaneously over a period of 6 weeks. Previous studies demonstrated that anemia of inflammation caused by iron sequestration and impaired erythropoiesis in the setting of acute or chronic inflammation was an independent predictor of increased mortality in critically ill patients [17–19]. Hence, the lower the hemoglobin level is in the acute phase of KD, the more severe the coronary artery damage will be. Hemoglobin during the acute phase, especially on the 5th day, can be used for risk stratification in infants with KD.

There are some limitations to our study. This was a single-center retrospective study. Therefore, further multicenter studies with adequate sample sizes are warranted to assess the generalizability of these findings. In addition, we analyzed only blood cell counts and CRP as other laboratory values were not available for all 2 selected time points. Nevertheless, the laboratory markers we analyzed in this study are the most readily available and inexpensive parameters. Last, the acute phase treatment was not standardized, and the coronary Z-score was not based on the normal value for Chinese children.

In conclusion, incomplete KD and CAA occurred frequently in infants. Peak percentage neutrophils within 2–5 days of illness and peak platelet count within 8–10 days of illness were significantly associated with the risk of CAA. Hemoglobin within 4–6 days was associated with persistent CAA and may be used as a predictor of persistent CAA due to KD in infants. For infants with high peak percentage neutrophils within 2–5 days of illness, the use of steroids, cyclosporine, or infliximab in combination with initial IVIG should be considered in the early stages of KD.

## Data Availability

The datasets used and analysed during the current study available from the corresponding author on reasonable request.
